# Utilization of rye as energy source affects bacterial translocation, intestinal viscosity, microbiota composition, and bone mineralization in broiler chickens

**DOI:** 10.3389/fgene.2014.00339

**Published:** 2014-09-25

**Authors:** Guillermo Tellez, Juan D. Latorre, Vivek A. Kuttappan, Michael H. Kogut, Amanda Wolfenden, Xochitl Hernandez-Velasco, Billy M. Hargis, Walter G. Bottje, Lisa R. Bielke, Olivia B. Faulkner

**Affiliations:** ^1^The John Kirkpatrick Skeeles Poultry Health Laboratory, Department of Poultry Science and The Center of Excellence for Poultry Science, University of ArkansasFayetteville, AR, USA; ^2^Southern Plains Area Home, United States Department of Agriculture – Agricultural Research Service, College StationTX, USA; ^3^Facultad de Medicina Veterinaria y Zootecnia, Universidad Nacional Autónoma de MéxicoMexico City, México

**Keywords:** bacterial translocation, intestinal viscosity, rye, bone mineralization, chickens

## Abstract

Two independent trials were conducted to evaluate the utilization of rye as energy source on bacterial translocation (BT), intestinal viscosity, gut integrity, gut microbiota composition, and bone mineralization, when compared with a traditional cereal (corn) in broiler chickens. In each experiment, day-of-hatch, broiler chickens were randomly assigned to either a corn or a rye diet (*n* = 20 chickens/group). At 10 d of age, in both experiments, 12 chickens/group were randomly selected, and given an oral gavage dose of fluorescein isothiocyanate dextran (FITC-d). After 2.5 h of oral gavage, blood samples were collected to determine the passage of FITC-d. The liver was collected from each bird to evaluate BT. Duodenum, ileum, and cecum gut sections were collected to evaluate intestinal viscosity and to enumerate gut microbiota. Tibias were collected for observation of bone parameters. Broilers fed with rye showed increased (*p* < 0.05) intestinal viscosity, BT, and serum FITC-d. Bacterial enumeration revealed that chickens fed with rye had increased the number of total lactic acid bacteria in all three sections of the gastrointestinal tract evaluated when compared to chickens fed with corn. Chickens fed with rye also had significantly higher coliforms in duodenum and ileum, whereas the total number of anaerobes increased only in duodenum. A significant reduction in bone strength and bone mineralization was observed in chickens fed with rye when compared with corn fed chickens. In conclusion, rye evoked mucosal damage in chickens that alter the intestinal viscosity, increased leakage through the intestinal tract, and altered the microbiota composition as well as bone mineralization. Studies to evaluate dietary inclusion of selected DFM candidates that produce exogenous enzymes in rye fed chickens are currently being evaluated.

## INTRODUCTION

The intestinal epithelium constitutes the largest and most important barrier against external environmental agents and has two critical functions: to prevent the entry of harmful intraluminal microorganisms, antigens, and toxins, and to enable the selective translocation of dietary nutrients and electrolytes into circulation (Salminen and Isolauri, 2006; Salzman, 2011; Elson and Cong, 2012). One of the basic properties of gut-associated lymphoid tissue (GALT) is oral tolerance (unresponsiveness) to harmless components of microbiota and diet (Kau et al., 2011). Inappropriate immunological reactions against food compounds, such as lactose or gluten, can lead to the breakdown of oral tolerance and the development of intestinal immune disorders (Marsh, 1992; Stepniak and Koning, 2006). For example, Celiac disease (CD) is a chronic immune-mediated enteropathy of the small intestine that is triggered by dietary wheat gluten, or related rye and barley proteins in genetically susceptible individuals (Williamson and Marsh, 2002). The clinical and pathological spectrum of CD is heterogeneous and there is no current rodent model that reproduces all aspects of human CD (Schuppan et al., 2009; Kupfer and Jabri, 2012). Patients display intestinal barrier dysfunction and altered tight junction protein expression allowing abnormal penetration of gluten-related peptides and enteric microbes, which could stimulate any subsequent immune response (Silva et al., 2012; Ströhle et al., 2013). Clinical presentation of CD can vary from a classical malabsorption syndrome to more subtle atypical gastrointestinal manifestations (similar to irritable bowel syndrome) or extra intestinal presentations (infertility, osteoporosis, and iron-deficiency anemia; James, 2005; Bianchi and Bardella, 2008; Bianchi, 2010; Assimakopoulos et al., 2011; Leﬄer, 2011). Likewise, the composition of the diet, also has a tremendous impact in digestibility and gut health of chickens (Dunsford et al., 1989; Hrncir et al., 2008; Maslowski and Mackay, 2011). A specific example is rye-based diets versus traditional corn-based diets, where different cereals are used as the principal source of energy. The inclusion of rye in poultry diets has been fraught with problems, principally related to the production of sticky droppings, malabsorption syndrome, elevated feed conversion, and intestinal bacterial overgrowth (Campbell et al., 1983; Bedford and Schulze, 1998; Shirzadi et al., 2010). The purpose of the present study was to evaluate the utilization of rye as energy source on bacterial translocation (BT), intestinal viscosity, microbiota composition and bone mineralization when compared with a traditional cereal (corn) in broiler chickens.

## MATERIALS AND METHODS

### ANIMAL SOURCE AND EXPERIMENTAL DESIGN

In order to show that the same or similar results can be achieved independently, two experiments were conducted in the present study. In each experiment, forty broiler chickens were obtained from Cobb-Vantress (Siloam Springs, AR, USA); the number of animals used was based on published studies in which similar outcomes were measured (Campbell et al., 1983; Zhang and Coon, 1997; Higgins et al., 2010a,b, 2011). Chickens were randomly assigned to two groups (*n* = 20 chickens), and placed into isolator chambers in a controlled age-appropriate environment with unrestricted access to feed and water for 10 days. All animal handling procedures were in compliance with Institutional Animal Care and Use Committee at the University of Arkansas. At 10 days of age, in both experiments, 12 chickens in both treatment groups were randomly selected, and given an oral gavage dose of fluorescein isothiocyanate dextran (FITC-d; 2.2 mg/mL/bird; MW 3,000–5,000 Da; Sigma Aldrich Co., St. Louis, MO, USA). After 2.5 h they were humanly killed. Blood samples were collected from the femoral vein for measuring leakage of FITC-d. Liver was collected to evaluate BT. Duodenal, ileal, and cecum gut sections were collected to enumerate bacteria. For intestinal viscosity, five chickens from each group were humanly killed and intestinal digesta were individually collected. Additionally, tibias were collected for bone parameters as describe below.

### DIETS

The rye used in this research contained: 2710 Kcal of metabolizable energy, 12.6% of crude protein, 2.8% of crude fiber, 0.40% of Lysine, 0.16% of methionine, 0.08% of calcium, and 0.3% of phosphorus. In the case of corn it contained: 3350 Kcal of metabolizable energy, 8.27% of crude protein, 0.24% of Lysine, 0.15% of methionine, 0.01% of calcium, and 0.28% of phosphorus (**Table 1**). The differences in the nutritional composition of the two cereals used as principal sources of energy explain the variation between the experimental diets, which were formulated to approximate the nutritional requirements proposed by the National Research Council (). Due to a lower energetic contribution of rye, it was required to increase the percentage of inclusion of fat in this diet. On the contrary, in the case of crude protein in the corn diet, it was required to use a higher amount of soybeans in the feed formula to fulfill the nutritional requirements, because of the minor contribution of corn as a protein source. The small difference showed in the percentage of inclusion of lysine (L-Lysine HCL) and methionine (DL-Methionine) as purified amino acids in the diets is due to differences in amount and nutritional composition of soybean, corn, and rye in used in each diet (**Table 1**). However, both diets satisfy the amino acid requirements for maintenance and growth according to age, sex, and genetic line of the animals used in the present experiment. Broilers in the starter period (0–10 days of age) required between 21 and 22% of crude protein, 3035 Kcal of metabolizable energy, 1.32% of Lysine, 0.50% of Methionine, 0.90% of Calcium, and 0.45% of available phosphorus, all this specifications were taken into account to formulate and prepare the diets used in the present study.

**Table 1:** Ingredients (%) of the corn and rye diets used in Experiments 1 and 2.

Item	Corn diet	Rye diet
**Ingredients**
Corn	55.53	0.0
Rye	0.00	**58.27**
Soybean meal	**35.69**	**31.16**
Vegetable oil	4.22	6.29
Dicalcium phosphate	1.82	1.79
Calcium carbonate	1.12	1.05
Salt	0.38	0.38
DL-METHIONINE	0.37	0.35
Vitamin premix^1^	0.20	0.20
L-LYSINE HCL	0.28	0.22
Choline chloride 60%	0.20	0.10
Mineral premix^2^	0.10	0.10
Selenium 0.6%	0.02	0.02
Propionic acid	0.02	0.02
Antioxidant^3^	0.05	0.05
Total	100.00	100.0

*^1^Vitamin premix supplied the following per kg: vitamin A, 20,000,000 IU; vitamin D3, 6,000,000 IU; vitamin E, 75,000 IU; vitamin K3, 9 g; thiamine, 3 g; riboflavin, 8 g; pantothenic acid, 18 g; niacin, 60 g; pyridoxine, 5 g; folic acid, 2 g; biotin, 0.2 g; cyanocobalamin, 16 mg; and ascorbic acid, 200 g (Nutra Blend LLC, Neosho, MO 64850, USA).*

*^2^Mineral premix supplied the following per kg: manganese, 120 g; zinc, 100 g; iron, 120 g; copper, 10–15 g; iodine, 0.7 g; selenium, 0.4 g; and cobalt, 0.2 g (Nutra Blend LLC, Neosho, MO 64850, USA).*

*^3^Ethoxyquin.*

### VISCOSITY

Total intestinal digesta contents were collected from Meckel’s diverticulum to the ileocecocolonic junction. For viscosity analysis, approximately 1.5 g (wet weight) of the fresh digesta was immediately placed in a microcentrifuge tube and centrifuged at 12,000×*g* at 4^∘^C for 5 min. The supernatant fluid was collected and stored on ice until viscosity measurement was determined using a LVDV-I Brookfield digital cone-plate viscometer fitted with a CP-40 spindle (Brookfield Engineering, Middleboro, MA, USA). The analyzed samples and the viscometer cup were maintained at 40^∘^C during viscosity measurement. Viscosity was measured in centipoise (cP = 1/100 dyne s/cm^2^) and the results were reported as log_10_ cP.

### BACTERIAL TRANSLOCATION

Briefly, the right half of the liver was removed from each chicken, collected in sterile bags, homogenized, weighed and 1:4 wt/vol dilutions were made with sterile 0.9% saline. Ten-fold dilutions of each sample, from each group were made in a sterile 96 well Bacti flat bottom plate and the diluted samples were plated on MacConkey Agar (VWR Cat. No. 89429-342 Suwanee, GA 30024, USA). Biochemical evaluation tests as well as identification of isolated colonies were carried out using a bioMerieux API 50 CH test kit (catalog no. 50430, bioMerieux, Marcy l’Etoile, France).

### SERUM DETERMINATION OF FITC-d

Blood was kept at room temperature for 3 h and centrifuged (1,000×*g* for 15 min) to separate the serum from the red blood cells. FITC-d levels of undiluted serum were measured at excitation wavelength of 485 nm and emission wavelength of 528 nm (Synergy HT, Multi-mode microplate reader, BioTek Instruments, Inc., Vermont, USA). Fluoroscence measured was then compared to a standard curve with known FITC-d concentrations. Gut leakage for each bird was reported as μg of FITC-d/mL of serum.

### ENUMERATION OF BACTERIA

Whole duodenum, ileum, and both ceca were aseptically removed, separated into sterile bags, and homogenized. Samples were weighed and 1:4 wt/vol dilutions were made with sterile 0.9% saline. Ten-fold dilutions of each sample, from each group were made in a sterile 96 well Bacti flat bottom plate and the diluted samples were plated on three different culture media; to evaluate total number of lactic acid bacteria (LAB) in Man Rogosa Sharpe (Difco Lactobacilli MRS Agar VWR Cat. No. 90004-084 Suwanee, GA 30024, USA); total Enterobacteriaceae in MacConkey; and total anaerobes in tryptic soy agar with sodium thioglycolate plates (Tryptic Soy Agar, catalog no. 211822, Becton Dickinson, Sparks, MD, USA).

### BONE PARAMETERS

Bone parameters were measured according to the methods as described by Zhang and Coon (1997). Tibias from each chicken were cleaned of attached tissues. Bones from the left leg were subjected to conventional bone assays and tibia from the right leg was used to determine breaking strength. The bones from left tibia were dried at 100^∘^C for 24 h and weighed again. The samples were then incinerated in a muﬄe furnace (Isotemp muﬄe furnace, Fisher Scientific, Pittsburgh, PA, USA) at 600^∘^C for 24 h in crucibles. Finally, the content of calcium and phosphorus in the tibia was determined using standard methods (AOAC International, 2000) and were reported as percentage of dry matter. The right tibial diaphyses from individual birds were cleaned of adherent tissues, the periosteum was removed, and the biomechanical strength of each bone was measured using an Instron 4502 (Norwood, MA, USA) material testing machine with a 100 kg Load Cell. The bones were held in identical positions and the mid-diaphyseal diameter of the bone at the site of impact was measured using a dial caliper. The maximum load at failure was determined using a three-point flexural bend fixture with a total distance of 30 mm between the two lower supporting ends. The load, defined as force in kilograms per square millimeter of cross-sectional area (kg/mm^2^), represents bone strength. The rate of loading was kept constant at 20 mm/min collecting 10 data points per second. The data were automatically calculated using Instron’s Series IX Software (Norwood, MA, USA).

### STATISTICAL ANALYSIS

All data were subjected to one-way analysis of variance as a completely randomized design using the General Linear Models procedure of SAS (SAS Institute, 2002). Data are expressed as mean ± standard error. Significant differences among the means were determined by using Duncan’s multiple-range test at *p* < 0.05.

## RESULTS

The evaluation of body weight, intestinal viscosity, serum FITC-d, and liver BT in broiler chickens fed with a corn diet or a rye diet of Experiment 1 and 2 are summarized in **Table 2**. A significant (*p* < 0.05) reduction in body weight was observed in chickens fed with rye as compared with corn in both experiments. However, chickens fed with rye showed a increase in intestinal viscosity which was associated with elevated (*p* < 0.05) serum FITC-d, and an increase in BT to the liver (**Table 2**). Identification of the Gram-negative lactose positive bacteria in the liver was confirmed to be *Escherichia coli*.

**Table 2:** Evaluation of body weight, intestinal viscosity, serum FITC-d, and liver bacterial translocation in chickens fed with corn or rye in Experiments 1 and 2.

	**Body weight(g)**	**Intestinal viscosity (cP Log_**10**_)**	**Serum FITC-d (μg/mL)**	**Bacterial translocation (CFU Log_**10**_)**
**Experiment 1**
**Corn**	283.21 ± 10.57^a^	0.30 ± 0.04^b^	0.20 ± 0.01^b^	0.00 ± 0.00^b^
**Rye**	110.69 ± 5.21^b^	2.84 ± 0.57^a^	0.42 ± 0.05^a^	1.35 ± 0.45^a^
**Experiment 2**
**Corn**	301.46 ± 10.57^a^	0.23 ± 0.35^b^	0.31 ± 0.03^b^	0.00 ± 0.00^b^
**Rye**	140.89 ± 5.21^b^	2.90 ± 0.83^a^	0.52 ± 0.07^a^	2.40 ± 0.73^a^

*Data is express as Mean ± SE. Intestinal viscosity is expressed in Log_10_ (in centipoise, cP = 1/100 dyne s/cm^2^) from five chickens. Serum FITC-d and liver BT (expressed in cfu Log_10_/g of tissue) from 12 chickens. ^a,b^within columns indicate significant difference at *p* < 0.05.*

Total bacterial counts in duodenum, ileum, and ceca of chickens fed with a corn or rye diet in Experiments 1 and 2 are summarized in **Table 3**. In both trials, chickens that were fed with rye had a significant increase in the number of total LAB that were observed in the duodenum, in the ileum, and in the ceca when compared with chickens fed with corn. In these chickens, a significant increase in the total number of coliforms was also observed in duodenum and ileum but not in cecum, whereas, an increase in total number of anaerobes was observed only in the duodenum.

**Table 3:** Evaluation of total bacterial counts in duodenum, ileum, or cecum in chickens fed with corn or rye in Experiments 1 and 2.

	Duodenum			Ileum			Ceca		
	Coliforms	LAB’s	Anaerobes	Coliforms	LAB	Anaerobes	Coliforms	LAB	Anaerobes
**Experiment 1**
**Corn**	1.0 ± 0.19^b^	2.29 ± 0.76^b^	3.86 ± 0.40^b^	1.92 ± 0.11^b^	3.98 ± 0.58^b^	5.16 ± 0.11^a^	7.75 ± 0.14^a^	7.02 ± 0.14^b^	7.91 ± 0.20^a^
**Rye**	3.62 ± 0.35^a^	5.91 ± 0.25^a^	5.36 ± 0.31^a^	4.87 ± 0.70^a^	6.25 ± 0.57^a^	5.41 ± 0.50^a^	7.48 ± 0.15^a^	8.02 ± 0.14^a^	7.83 ± 0.30^a^
**Experiment 2**
**Corn**	2.27 ± 0^b^	3.27 ± 0.76^b^	3.27 ± 0.40^b^	1.42 ± 0.11^b^	3.42 ± 0.58^b^	3.42 ± 0.11^a^	8.08 ± 0.14^a^	7.08 ± 0.14^b^	8.08 ± 0.20^a^
**Rye**	3.77 ± 0.35^a^	5.77 ± 0.25^a^	6.23 ± 0.31^a^	3.66 ± 0.70^a^	6.66 ± 0.57^a^	3.66 ± 0.50^a^	7.71 ± 0.15^a^	7.71 ± 0.14^a^	7.71 ± 0.30^a^

*Data is express in Log_10_ cfu/g of tissue. Mean ± SE from 12 chickens. LAB. ^a,b^within columns indicate significant difference at p < 0.05.*

The results of the evaluation of bone breaking strength and bone parameters in chickens fed with corn or rye in Experiments 1 and 2 are summarized in **Table 4**. Significant increases in tibia diameter, tibia breaking strength, tibia ash, and calcium and phosphorus percentages were observed in chickens that fed corn when compared with chickens that fed the rye diet (**Table 4**).

**Table 4:** Evaluation of bone breaking strength and bone composition parameters in chickens fed with corn or rye based diets in Experiments 1 and 2.

	Tibia strength Load at yield (kg/mm^2^)	Tibia diameter (mm)	Total ash from tibia (%)	Calcium (% of ash)	Phosphorus (% of ash)
**Experiment 1**
**Corn**	5.04 ± 0.011^a^	3.34 ± 0.17^a^	55.01 ± 0.41^a^	29.48 ± 0.27^a^	18.15 ± 0.12^a^
**Rye**	1.58 ± 0.009^b^	1.61 ± 0.28^b^	34.87 ± 0.35^b^	18.48 ± 0.27^b^	13.15 ± 0.12^b^
**Experiment 2**
**Corn**	6.14 ± 0.01^a^	4.55 ± 0.32^a^	65.61 ± 0.81^a^	37.65 ± 0.07^a^	21.35 ± 0.52^a^
**Rye**	2.58 ± 0.03^b^	1.82 ± 0.78^b^	30.87 ± 0.75^b^	21.32 ± 0.46^b^	15.67 ± 0.29^b^

*Tibias from 12 chickens were collected to evaluate bone quality. Data is expressed as mean ± standard error. ^a,b^within columns indicate significant difference at *p* < 0.05.*

## DISCUSSION

In recent years, nutrition research has moved from classical epidemiology and physiology to molecular biology and genetics. Modern nutritional research is aimed at health promotion, at disease prevention, and on performance improvement (Kussmann et al., 2008). Hence, nutritional sciences are discovering the application of the so called “omics” sciences (Ghosh and Poisson, 2009; Dimitrov, 2011). Nutritional genomics is a recent off-shoot of this genetic revolution. As a consequence of these ambitious objectives, the disciplines “nutrigenetics” and “nutrigenomics” have evolved (Afman and Müller, 2006). in particular, nutrigenomics, is the junction between health, diet, and genomics, it can be seen as the combination of molecular nutrition and genomics, addressing the inverse relationship, which is how diet influences gene transcription, protein expression, and metabolism. (Davis and Milner, 2004; Trujillo et al., 2006; Wishart, 2008; García-Cañas et al., 2010). With emerging “omics” technologies, scientists are now better able to investigate how dietary food components can affect physiological functions and the underlying cellular and molecular mechanisms implicated in the digestive process (Ghosh and Poisson, 2009; Dimitrov, 2011). Nutrition-related genomics technology has revolutionized the field of nutrition providing unprecedented opportunities for increasing our understanding of how nutrients modulate gene and protein expression to influence cellular metabolism (Davis and Milner, 2004; Trujillo et al., 2006; Subbiah, 2007; Wishart, 2008; García-Cañas et al., 2010).

When integrated with other “omics” technologies in a biological system approach, novel nutrition-based intervention strategies are expected to provide an effective alternative for disease control. Several studies in poultry have been conducted to investigate the effects of probiotics and phytonutrients on the translational regulation of genes associated with immunology, pathogen control, physiology, and metabolism using high-throughput microarray analysis and *in vivo* disease challenge models (Tellez et al., 1993; Higgins et al., 2007, 2008, 2010a,b, 2011; Timbermont et al., 2010; Lillehoj et al., 2011; Jerzsele et al., 2012; Kiarie et al., 2013; Layton et al., 2013). Chicken has been an important experimental system for developmental biology, immunology, and microbiology for more than 2 millennia (Stern, 2004) having led to many fundamental discoveries (Burt, 2007). With the evolution of sequencing of the genome and the development of high-throughput tools for the exploration of functional elements of the genome, chicken has attained a model organism status (Stern, 2005; Dodgson, 2007). The increase in genomic resources, easy access to the embryo, and the application of RNA interference showed that it will be easy and quick to use chicken embryos to screen the function of many genes during embryonic development (Stern, 2004). So, it seems likely that chicken will increasingly be the system of choice for many vertebrate biologists, especially in the field of human sciences, who are interested in gene function (Aggarwal and Lee, 2003; Hillier et al., 2004; Cogburn et al., 2007).

Thousands of years of evolution shaped the digestive system of the jungle fowl and wild pig to deal with the dietary ingredients they encounter in an efficient manner. More recently, throw intensive genetic manipulation, nutrition, and health programs we have altered the biology and growth potential of chickens and pigs among other productive animals (Muramatsu et al., 1990; Fuller et al., 1995). In the wild, the diets of these animals would be made up of many different ingredients, few of which would ever reach>30% of total intake on a lifetime basis. The range in types and relative quantities of ingredients that can be presented to the modem commercial monogastric animals, while complex, tend to result in a diet in which two or three ingredients may constitute >75% of intake (National Research Council, 1994). Such change is driven by least cost formulation processes, and endeavors to provide maximum nutrient density for minimum cost (Bedford and Schulze, 1998). Corn is usually the main source of energy in poultry diets, but at times it is difficult to formulate least cost diets using corn and unconventional grains have to be used. When chickens are fed alternative grains such wheat or rye that are high in non-starch polysaccharides (NSPs), poor performance and unmanageable litter conditions caused by sticky droppings are reported (Campbell et al., 1983; Fengler and Marquardt, 1988; Choct et al., 1995). Wheat or rye contains high concentrations of NSPs, leading to reduced digestibility. In addition, high NSPs diets have also been associated with Necrotic Enteritis (NE), a multi-factorial disease caused by *Clostridium perfringens* (CP) that is probably the most important bacterial disease in terms of economic implications in broiler chickens (Hofacre, 2001; Annett et al., 2002; Timbermont et al., 2011). NSPs, are comprised mainly of highly branched arabinoxylans, increassig digesta viscosity responsible for poor digestibility through interference with the movement of particles and solutes across the intestinal lumen, preventing the access of digestive enzymes to the endosperm contents and reducing intestinal absorption of sodium and calcium (Fengler and Marquardt, 1988). In addition, increased digesta viscosity reduce conjugated bile acid, affecting fat emulsification and fat digestibility (Langhout et al., 1997). In the present study, the significant reduction in bone strength and mineralization (**Table 4**) confirmed previous studies that have shown that high NSPs diets in poultry or gluten intolerance in humans, are also associated with malabsorption of minerals and fat-soluble vitamins (MacAuliffe and McGinnis, 1971; Campbell et al., 1983; Rennie et al., 1993; Bianchi and Bardella, 2008; Capriles et al., 2009; Kotake et al., 2009; Wideman and Prisby, 2011). In our two experiments, the viscosity of the gut in chickens fed with rye was so extreme, the supernatant being more semi-solid than fluid, that it alone could be directly responsible for the poor performance (**Table 2**). Condensed feed passage rate increases the time available for digesta associated bacteria to multiply prior to evacuation in the feces, and provides more substrate availability in the distal parts of the small intestine for microbial fermentation (Choct et al., 1995; Murphy et al., 2009; Kiarie et al., 2013). In the present study, the increase intestinal viscosity observed in chickens fed with rye was also associated with elevated BT, increased serum FITC-d and bacterial overgrowth when compared with chickens fed with corn (). Variations in the composition of the microbiome within different segments of the alimentary tract are influenced by the environment, by the diet, and by the host (Ouwehand et al., 2002; Xu and Gordon, 2003; Hooper, 2004; Mazmanian et al., 2008). Alterations in gut permeability are connected with BT in the portal and/or systemic circulation in several types of leaky gut syndromes leading to systemic bacterial infections (Ilan, 2012; Seki and Schnabl, 2012). Similarly, FITC-d is a large molecule (3–5 kDa) which does not usually leak through the intact gastrointestinal tract barrier. However, when conditions disrupt the tight junctions between epithelial cells, the FITC-d molecule can enter circulation as demonstrated by an increase in trans-mucosal permeability associated with chemically induced disruption of tight junctions by elevated serum levels of FITC-d after oral administration (Yan et al., 2009). Since poultry has little or no intrinsic enzymes capable of hydrolyzing these NSPs, exogenous xylanases as additives are used in an attempt to reduce this anti-nutritive factors (Bedford et al., 1991; Bedford and Classen, 1993; Bedford and Schulze, 1998). Previously, we have evaluated the inclusion of selected direct-fed microbial (DFM) candidates that produce exogenous enzymes (protease, phytase, lipase, xylanase, and cellulose) in high NSPs diets (rye, wheat, barley, and oat). In those studies, a significant reduction in both viscosity and *C. perfringens* proliferation was observed between high NSPs control non-treated diets or the same diets supplemented with DFM *in vitro* (Latorre et al., 2014; Tellez et al., 2014). The results of the present study have confirmed in some extent our earlier *in vitro* findings. Together, they represent a step toward the application of nutrigenomics in the context of a chicken model. The incorporation of one or more “omics” techniques (in particular, assessment of the microbiome) will provide a better understanding of how dietary food components can affect physiological functions and the fundamental cellular and molecular mechanisms implicated in the digestive process of high NSPs diets in chickens.

In conclusion, the use of rye as an energy source increased intestinal viscosity, increased BT, and leakage of FITC-d, altered the microbiota composition as well as bone mineralization in chickens. Studies to evaluate dietary inclusion of selected DFM candidates that produce exogenous enzymes in rye fed chickens are currently being evaluated.

## Conflict of Interest Statement

The authors declare that the research was conducted in the absence of any commercial or financial relationships that could be construed as a potential conflict of interest.
